# Antioxidant Food Components for the Prevention and Treatment of Cardiovascular Diseases: Effects, Mechanisms, and Clinical Studies

**DOI:** 10.1155/2021/6627355

**Published:** 2021-01-28

**Authors:** Dan-Dan Zhou, Min Luo, Ao Shang, Qian-Qian Mao, Bang-Yan Li, Ren-You Gan, Hua-Bin Li

**Affiliations:** ^1^Guangdong Provincial Key Laboratory of Food, Nutrition, and Health, Department of Nutrition, School of Public Health, Sun Yat-sen University, Guangzhou 510080, China; ^2^Research Center for Plants and Human Health, Institute of Urban Agriculture, Chinese Academy of Agricultural Sciences, Chengdu 610213, China

## Abstract

Cardiovascular diseases (CVDs) have gained increasing attention because of their high prevalence and mortality worldwide. Epidemiological studies revealed that intake of fruits, vegetables, nuts, and cereals could reduce the risk of CVDs, and their antioxidants are considered as the main contributors. Moreover, experimental studies showed that some antioxidant natural products and their bioactive compounds exerted beneficial effects on the cardiovascular system, such as polyphenols, polysaccharides, anthocyanins, epigallocatechin gallate, quercetin, rutin, and puerarin. The mechanisms of action mainly included reducing blood pressure, improving lipid profile, ameliorating oxidative stress, mitigating inflammation, and regulating gut microbiota. Furthermore, clinical trials confirmed the cardiovascular-protective effect of some antioxidant natural products, such as soursop, beetroot, garlic, almond, and green tea. In this review, we summarized the effects of some antioxidant natural products and their bioactive compounds on CVDs based on the epidemiological, experimental, and clinical studies, with special attention paid to the relevant mechanisms and clinical trials.

## 1. Introduction

Cardiovascular diseases (CVDs), such as coronary heart disease (CHD), hypertensive heart disease, heart failure, and stroke, are the leading cause of death worldwide [1]. CVDs could be caused by hypertension, dyslipidemia, atherosclerosis, oxidative stress, inflammation, and enteric dysbacteriosis [2–4]. Several synthetic drugs have been used to treat CVDs, but they showed some adverse effects, such as gastrointestinal reaction, hyperkalemia, and arrhythmias [5, 6]. On the other hand, accumulating evidence has shown that some antioxidant natural products could be a safe and effective alternative for the prevention and treatment of CVDs [7–11].

Natural products are rich in dietary fibers, polyphenols, vitamins, minerals, and other beneficial components, and possess many bioactivities, such as antioxidant, anti-inflammatory, anticancer, antidiabetic, antiobesity, hepatoprotective, immunoregulatory, antibacterial, and cardiovascular-protective effects [12–20]. Epidemiological studies found that people consuming more fruits, vegetables, teas, cereals, and nuts had a lower risk of CVDs, and the antioxidants in these natural products were considered as the main contributors [21–23]. Additionally, experimental researches showed that some antioxidant natural products and their active compounds could prevent and treat CVDs through different mechanisms of action [24–32]. Furthermore, clinical trials provided more reliable human evidence on some antioxidant natural products for the prevention and treatment of CVDs [33, 34]. The purpose of this review is to summarize the effect of some antioxidant natural products and their bioactive compounds on CVDs from the results of epidemiological, experimental and clinical studies in the last five years, and special attention was paid to the mechanisms of action and clinical trials.

## 2. Epidemiological Studies

Increasing epidemiological studies have suggested that the intake of some antioxidant natural products significantly attenuated the risk factors of CVDs (Table 1).

Several cross-sectional studies found that some dietary plants were beneficial for the prevention and management of CVDs. For instance, a cross-sectional study of 18,757 Chinese adolescents aged 13-17 years revealed that daily intake of at least 3 servings of vegetables (1 serving of vegetable was the size of an adult's fist) lowered the risk of hypertension (odds ratio (OR) = 0.74; 95% confidence interval (CI): 0.42-0.95) compared with daily consumption of vegetable <1 serving [35]. Additionally, an analysis of 18,433 American adults found that compared with the lowest tertile consumption of cereals, vegetables, and fruit fibers as well as their total fiber, the OR (95% CI) of hypertension for the highest tertile were 0.80 (0.69-0.98), 0.82 (0.69-0.98), 0.86 (0.71-1.04), and 0.62 (0.52-0.75), respectively, indicating that cereals, vegetables, and total fibers were inversely related with hypertension, but fruit fiber was not [36]. Moreover, the data from the Korea National Health and Nutrition Examination Survey showed that overweight older males and younger females who consumed a moderate amount of curry (2-3 times a month or once a week), mainly composed of turmeric, had significantly lower levels of blood glucose and triglyceride (TG) than a group who had low curry consumption (almost never, or once a month) [37].

A negative correlation between the intake of several edible plants and the incidence as well as mortality of CVDs was also observed in some cohort studies. A follow-up study recruiting 521,891 Chinese adults aged 30-79 years reported that participants who consumed fresh fruit daily had lower systolic blood pressure (SBP) and blood glucose level compared to those who never or rarely ate fresh fruit. The HR (95% CI) for cardiovascular death, incident major coronary events, ischemic stroke, and hemorrhagic stroke were 0.60 (0.54-0.67), 0.66 (0.58-0.75), 0.75 (0.72-0.79), and 0.64 (0.52-0.74), respectively, elucidating the protective effect of fresh fruit on the cardiovascular system [38]. Moreover, another analysis of 3,052 adults indicated that the habitual consumption of allium vegetables, such as garlic and onion, was related to a 64% decreased risk of CVD outcomes (HR = 0.36; 95% CI: 0.18-0.71) [39]. Additionally, a cohort study of young Mediterranean populations found that compared to the lowest quintile of fruit intake or whole grain intake, the HR (95% CI) of the risk of CVD events for the highest quintile were 0.51 (0.27-0.95) and 0.43 (0.20-0.93), respectively, showing the benefits of fruit or whole grain to prevent CVDs [40]. Furthermore, a prospective study of 2,295 Iranian adults pointed out that compared to the lowest tertile of dietary fiber intakes from grains, legumes, nuts, fruits, and vegetables, the hazard ratios (HR) (95% CI) of CVD risks for the highest tertile were 0.90 (0.44-1.86), 0.31 (0.15-0.65), 0.49 (0.24-1.02), 0.44 (0.22-0.89), and 0.34 (0.16-0.72), respectively, suggesting that dietary fiber from legumes, fruits, and vegetables were negatively related to CVDs, while fiber from grains and nuts had no significant association with CVDs [41]. However, a cohort study found that nut intake significantly lowered the risk of CVDs [42]. The reason could be that it was not fiber but other bioactive compounds in nuts that play a vital role in the prevention of CVDs, or that there were disparities of population, study design, and confounding factors in different studies, which need to be further investigated in the future.

In short, the collected epidemiological investigations illuminated the protective effects of some antioxidant natural products and their bioactive components on CVDs, although there were inconsistent results. In addition, based on the beneficial role of some plants in CVDs, it is advisable to increase the intake of some plant-based foods, such as fresh fruits, vegetables, legumes, cereals, and nuts, to reduce the risk of CVDs.

## 3. Experimental Studies

**Figure 1:**
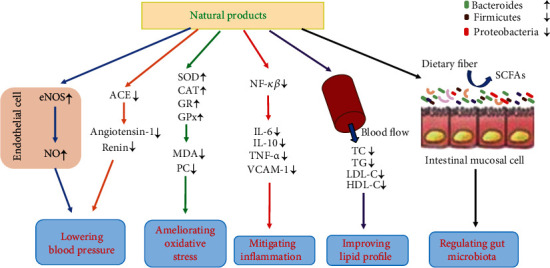
The mechanisms of natural products against cardiovascular diseases. Natural products could stimulate the activity of endothelial nitric oxide synthase (eNOS) and promote the release of nitric oxide (NO) to lower blood pressure; they can also inhibit the activity of angiotensin-1 converting enzyme (ACE) and decrease angiotensin-1 and renin to lower blood pressure. Natural products could promote the activities of antioxidant enzymes, like superoxide dismutase (SOD), catalase (CAT), glutathione reductase (GR), and glutathione peroxidase (GPx), and decrease the concentration of peroxidative products, like malondialdehyde (MDA) and protein carbonyls (PC), to ameliorate oxidative stress. Through the nuclear factor-kappa B (NF-*κβ*) signaling pathway, natural products could decrease levels of inflammatory markers, like tumor necrosis factor-*α* (TNF-*α*), interleukin-6 (IL-6), interleukin-10 (IL-10), and vascular cell adhesion molecule-1 (VCAM-1), to mitigate inflammation. Natural products could decrease the levels of total cholesterol (TC), triglyceride (TG), and low-density lipid protein cholesterol (LDL-C) and increase the level of high-density lipid protein cholesterol (HDL-C) to improve lipid profile. Natural products could increase the abundance of beneficial bacteria, like *Bacteroides*, and decrease the abundance of harmful bacteria, like *Firmicutes* and *Proteobacteria*, to regulate gut microbiota.

Many experimental studies investigated the effects of some antioxidant natural products and their bioactive compounds on CVDs (Table 2), and the relevant mechanisms are discussed below (Figure 1).

### 3.1. Reducing Blood Pressure

It's widely known that hypertension is an important risk factor for CVDs [56]. An analysis pointed out that every 10 mm Hg reduction in SBP markedly decreased the risk of major cardiovascular disease events in patients with a history of CVDs [57]. Some natural products are effective in the prevention and treatment of CVDs via reducing blood pressure. The hypotensive effect of these natural products was mainly related to the regulation of the renin-angiotensin system (RAS) and the release of nitric oxide (NO).

#### 3.1.1. Regulating the Renin-Angiotensin System

Blood pressure regulation is a sophisticated process involving various organs and systems, among which RAS plays an important role in elevating blood pressure [58]. Regulating the activity of RAS, such as inhibiting the synthesis of angiotensin-1 converting enzyme (ACE) as well as the secretion of renin/angiotensin, is helpful to ameliorate blood pressure [59, 60]. Many experimental studies revealed that some natural products performed the blood pressure lowering efficacy mainly though the regulation of RAS. For example, a study showed a potent *in vitro* ACE inhibitory property of winged bean seed hydrolysate, as well as the *in vivo* hypotensive effect of the hydrolysate in a dose-dependent manner in Sprague-Dawley (SD) rats, indicating that the hydrolysate lowered blood pressure via suppressing the activity of ACE [61]. Another study found that *Solanum macrocarpon* leaf extract suppressed the *in vitro* activities of renin and ACE. The oral administration of the extract decreased SBP, diastolic blood pressure (DBP), and heart rate in spontaneously hypertensive rats. Rutin, caffeic acid, and myricetin were the major polyphenols in the extract [62]. Furthermore, a study pointed out that *Ocimum sanctum* and *Citrus paradisi* infusions possessed a hypotensive property. The infusion of *Ocimum sanctum* downregulated the gene expression of renin and angiotensinogen and reduced renal triglyceride accumulation and lipid/protein oxidation in SD rats, while the hypotensive effect of *Citrus paradisi* could be associated with other mechanisms [63].

#### 3.1.2. Increasing the Release of NO

Accumulating evidence has proven that the generation of NO in endothelial cells is mainly activated by endothelial nitric oxide synthase (eNOS). NO could induce the relaxation of blood vessels, leading to the reduction of blood pressure [64, 65]. Hence, promoting the production of NO is an effective way to decrease blood pressure, which will protect the function of the cardiovascular system. There are findings suggesting that some natural products showed an antihypertensive effect via accelerating the release of NO, holding tremendous promise to prevent the development of hypertension and CVDs. For example, *Morus alba* induced endothelial vasorelaxation in mesenteric arteries via a NO-dependent pathway, and decreased blood pressure in wild-type mice. However, it failed to exert a hemodynamic effect in eNOS-deficient mice, which further testified to the antihypertensive action of *Morus alba* through a NO-dependent pathway [66]. Moreover, a study found that white mulberry fruit polysaccharides could provoke endothelium-dependent relaxation in rat mesenteric arteries and NO production in endothelial cells, and its intravenous injection induced the reduction of blood pressure in both normotensive rats and spontaneously hypertensive rats, while this effect was markedly attenuated in normotensive rats pretreated with the NO synthase inhibitor NG-nitro-L-arginine methyl ester (L-NAME). These results suggested that the hypotensive effect of white mulberry fruit was mediated by the NO pathway [67]. Additionally, grape seed polyphenol extract promoted the production of NO and reduced the blood pressure in hypertensive rats via upregulating the expression of eNOS and Sirtuin-1 [68].

### 3.2. Improving Lipid Profile

Hyperlipidemia results from the metabolic abnormalities of lipids, leading to higher levels of lipids in plasma than normal ones, which can be generally characterized as higher levels of total cholesterol (TC), triglyceride (TG), and low-density lipid protein cholesterol (LDL-C) and a lower level of high-density lipid protein cholesterol (HDL-C) [69]. Increasing evidence suggested that hyperlipidemia was closely associated with atherosclerosis, playing an important role in the development of CVDs [70, 71]. Several experimental studies revealed the hypolipidemic effect of natural products. For example, an *in vivo* study found that after the treatment of mung bean sprouts, the SBP and LDL-C levels of SD rats in the high-fat diet group significantly lowered to the normal level [72]. Additionally, supplementing obese rats with red dragon fruit flour for 4 weeks markedly reduced the blood glucose, TC, TG, and LDL-C levels, while HDL-C had no significant difference [73]. Also, another study found that after oral administration of red dragon fruit peel powder for 30 days, TC, TG, and LDL-C levels of hyperlipidemic male mice declined in a dose-dependent manner, accompanied by an increase in HDL-C levels [74]. The two studies above showed that both the pulp and peel of red dragon fruit possessed promising blood lipid-lowering efficacy. Furthermore, after administration with *Citrus maxima* juice for 14 days, male Wistar rats showed a significant decrease of TC and TG, along with an increase of HDL-C [75]. Furthermore, feeding with fresh bitter melon fruit juice for 4 weeks markedly dropped down the levels of blood glucose, TG, TC, and LDL-C in hyperglycemia rats compared with the initial levels, but the HDL-C level was dramatically elevated. Meanwhile, bitter melon effectively improved the fecal cholesterol secretion and suppressed cholesterol absorption, posing a potent ability to improve lipid profile [76].

### 3.3. Ameliorating Oxidative Stress

Oxidative stress, a major cause of the CVDs, is the result of the reduction of antioxidant capacity and the production of excessive reactive oxygen species (ROS) [77–79]. Some natural products could improve oxidative stress via promoting the activities of antioxidant enzymes, like superoxide dismutase (SOD), catalase (CAT), glutathione reductase (GR), and glutathione peroxidase (GPx) and decreasing the concentration of peroxidative products, like malondialdehyde (MDA) and protein carbonyls, hence promising to prevent and treat CVDs. A study showed that the intake of North American or Chinese wild rice effectively inhibited the formation of oxidative stress in hyperlipidemic rats via improving total antioxidant capacity, increasing SOD activity, and reducing MDA concentration. In addition, two wild rice varieties were also effective in suppressing hyperlipidemia and inflammation in rats [80]. Moreover, the polyphenol extract of *Sambucus nigra* L. ameliorated oxidative stress by enhancing total antioxidant capacity, and reduced both SBP and DBP in Wistar rats. Its combination with a renin inhibitor (Aliskiren) generated a superior antioxidant effect compared to administering the two separately, and it could also reduce the side effects of the antihypertensive agent [81]. Furthermore, the effects of dried chokeberry fruit extract on haemodynamic parameters, lipid profile, and oxidative stress were evaluated in spontaneously hypertensive rats, and anthocyanins, phenolic acids, and flavonoids in the extract were determined by the HPLC/DAD method. The extract rich in anthocyanins significantly reduced systolic and pulse pressures via increased diuresis. The thiobarbituric acid reactive substances (TBARS) in plasma and erythrocytes were significantly decreased in the treated group. The consumption of the extract also reduced lipid peroxidation through improving the ferric ion-reducing antioxidant power (FRAP) of plasma, but the activity of SOD in the treated group was significantly lower compared to the control group [26]. Additionally, the supplement of sweet cherry fruit and leaves to the high-fat–high-cholesterol diet in Wistar rats decreased body gain, improved liver function, and reduced inflammation and oxidative stress (by provoking the activities of SOD, GPx, GR, and CAT, and reducing the level of TBARS). The fruit and leaves reduced lipid accumulation in the liver and improved the lipid profile in serum. These effects could be from the regulation of the expression of fatty acid synthesis and oxidation-related genes [82]. In a previous study, the effects of rice bran protein hydrolysate on arterial stiffening, vascular remodeling, and oxidative stress were evaluated in rats fed a high-carbohydrate and high-fat diet. The hydrolysate supplementation significantly alleviated hyperglycemia, insulin resistance, dyslipidemia, hypertension, increased aortic pulse wave velocity, aortic wall hypertrophy, and vascular remodeling. The hydrolysate reduced the levels of ACE and tumor necrosis factor-alpha in plasma. The hydrolysate also alleviated oxidative stress by decreasing plasma MDA, reducing superoxide production, and suppressing p47 (phox) NADPH oxidase expression in the vascular tissues. The hydrolysate increased plasma nitrate/nitrite level and upregulated eNOS expression in the aortas of model group rats, indicating that the hydrolysate increased NO production [83]. In another study, *Zygophyllum album* root extract was analyzed using HPLC-DAD-ESI-QTOF-MS/MS, and twenty-six molecules were identified, including phenolic compounds and saponins. The extract significantly improved the heart injury markers, lipid peroxidation, protein oxidation, antioxidant capacity (SOD, CAT, and GPx), and DNA structure. The extract reduced the expressions of NF-kappa B, decreased plasmatic proinflammatory cytokine concentration, and suppressed the myocardial collagen deposition [84]. An *in vivo* study showed that apple polyphenol extract possessed a positive effect on vascular oxidative stress and endothelium function [85].

### 3.4. Mitigating Inflammation

Inflammatory response is a prominent pathological change in the development of CVDs, which can be characterized by increased levels of inflammatory markers, like tumor necrosis factor-*α* (TNF-*α*), interleukin-6 (IL-6), interleukin-10 (IL-10), C reactive protein (CRP), monocyte chemoattractant protein-1 (MCP-1), and vascular cell adhesion molecule-1 (VCAM-1) [86, 87]. It has been reported that some natural products were able to downregulate the expression of these cytokines and mitigate inflammation, which was a way of lowering the risk of CVDs. For example, an *in vivo* study pointed out that *Nepeta deflersiana* ethanolic extract effectively attenuated the myocardial injuries in Wistar rats by improving oxidative stress, inhibiting apoptosis, and mitigating inflammation. *Nepeta deflersiana* exerted an anti-inflammatory effect via the downregulation of the gene expression of TNF-*α*, IL-6, and IL-10 [29]. Another study found that the oral administration of *Zygophyllum album* root extract ameliorated the myocardial injuries in Wistar rats though the improvement of oxidative stress and the alleviation of inflammation. *Zygophyllum album* root extract decreased the plasma concentration of proinflammatory cytokines, like TNF-*α*, IL-1*β*, and IL-6 [84]. Additionally, spinach nitrate significantly lowered the elevated levels of serum CRP, TNF-*α*, and IL-6 induced by a high-fat and high-fructose diet in male mice, showing a strong anti-inflammatory capacity [88]. Also, a study demonstrated that *Spinacia oleracea* leaf methanolic extract dose-dependently attenuated isoproterenol-induced myocardial necrosis in male Wistar rats via mitigating the levels of proinflammatory cytokines, such as TNF-*α*, IL-1*β*, and IL-6 [89]. Moreover, *Antidesma bunius* extract significantly ameliorated the expressions of genes involved with proinflammatory cytokines, such as TNF-*α*, IL-6, VCAM-1, and MCP-1, showing great anti-inflammatory capacity [90].

### 3.5. Regulating Gut Microbiota

Recent interest has focused on the impact of gut microbiota on chronic diseases, especially CVDs. Increasing evidence has shown that gut microbiota was closely associated with the function of the cardiovascular system via contributing to the fermentation of dietary fiber in the colon, the production of short-chain fatty acids (SCFA), and the intestinal absorption of phytochemicals [91, 92]. Hence, it is of great significance to maintain the balance of intestinal flora to protect against CVDs. Some studies revealed that several natural products could regulate the homeostasis of gut microbiota. For example, a study demonstrated that wasabi powder prevented the development of hypertension in Wistar rats via changing the composition of gut microbiota, increasing the abundance of *Allobaculum*, *Sutterella*, *Uncl. S247*, *Uncl. Coriobacteriaceae*, and *Bifidobacterium* [93]. Moreover, treatment with anthocyanins extracted from *Lycium ruthenicum* Murray could not only improve oxidative stress and inflammation in C57BL/6 mice but also promote the proliferation of *Barnesiella*, *Alistipes*, *Eisenbergiella*, *Coprobacter*, and *Odoribacter* and increase the production of SCFA in cecal and feces [94]. Additionally, a study found that except for the improvement of lipid profile and inflammation, *Polygoni multiflori* Radix extract significantly inhibited atherosclerosis plaque formation in ApoE(-/-) mice via regulating gut microbiota composition [28]. Also, a study pointed out that tea polyphenols dose-dependently increased the abundance of intestinal *Bifidobacteria* in high-fat diet-fed ApoE(-/-) mice, and this increase negatively correlated with plaque area/lumen area ratios, suggesting that tea polyphenols could reduce atherosclerosis plaque induced by high-fat diet via increasing intestinal *Bifidobacteria* [95]. Furthermore, the intake of berry mixture, including blueberries, blackberries, raspberries, Portuguese crowberry, and strawberry tree fruit, increased the abundance of phylum *Bacteroidetes*, decreased the abundance of *Firmicutes*, and reduced the elevated abundance of *Proteobacteria* induced by a high-salt diet in Dahl salt-sensitive rats [96].

In brief, based on the *in vitro* and *in vivo* experimental studies, we summarized the potential mechanisms of some natural products protecting against CVDs, including reducing blood pressure, improving the lipid profile, ameliorating oxidative stress, mitigating inflammation, and regulating gut microbiota.

## 4. Clinical Trials

The benefits of reducing the risk of CVD events through the consumption of antioxidant natural products and their active ingredients have been studied in multiple clinical trials. Here, we summarize the protective effects of some natural products on CVDs (Table 3).

### 4.1. The Effects of Fruits on CVDs

Several clinical trials revealed the inverse relationship between the consumption of fruits and risk of CVDs. A randomized controlled trial (RCT) found that guava pulp significantly improved lipid profile by decreasing the levels of TC, TG, and LDL-C and increasing the level of HDL-C [101]. Another RCT pointed out that intake of 100 g soursop fruit twice per day for 3 months markedly decreased the levels of SBP, DBP, and serum uric acid in prehypertensive participants compared to the control group [102]. Additionally, a controlled nonrandomized clinical study showed that consuming 300 mL of orange juice for 2 months improved LDL-C, blood glucose, insulin sensitivity, and gut microbiota metabolism in healthy women [103]. Furthermore, a cross-over study found that after the intervention of 200 or 400 mg anthocyanin from haskap berry, the blood pressure levels in participants aged 62-81 years were significantly reduced [104]. Hence, consuming some fruits, like guava, soursop, and orange, is an effective way to prevent and manage CVDs.

### 4.2. The Effects of Vegetables on CVDs

Some vegetables also showed a protective effect on CVDs. An RCT found that the intake of 213 mg tomato extract for 4 weeks lowered DBP and mean arterial pressure in patients with hypertension and high risk of CVDs [105]. Moreover, several studies revealed the potent hypotensive efficacy of beetroot which was associated with its high content of nitrate. For example, a study demonstrated that consuming 70 mL beetroot juice significantly lowered the level of DBP in hypertensive pregnant women [106]. Additionally, daily consumption of 1.2 g eggplant powder markedly improved blood pressure and the psychological state of stressed participants with normal-high blood pressure or stage 1 hypertension [107]. Therefore, it is advisable to increase the intake of tomato, beetroot, and eggplant to protect the health of the cardiovascular system.

### 4.3. The Effects of Spices on CVDs

Similarly, some spices effectively reduced the risk of CVDs. An RCT showed that after intervention with 3 g cardamom for 2 months, the levels of TC and LDL-C in overweight and obese prediabetic women were remarkably lowered, while SBP, DBP, glycemic indices, and serum lipid values in the cardamom group did not significantly differ from the placebo group [108]. Another study compared the cardiovascular-protective effect of cardamom, cinnamon, saffron, and ginger, demonstrating that all of them showed potent abilities in controlling blood pressure and improving endothelial function [33]. Besides, daily ingestion of *Satureja hortensis* L. effectively improved the lipid profile in patients with metabolic syndrome by lowering TC, TG, and LDL-C and increasing HDL-C [109]. Moreover, garlic and cumin showed a strong hypotensive effect on patients with type 2 diabetes [110]. In brief, some spices, like cardamom, cinnamon, saffron, ginger, garlic, and cumin, hold great promise in preventing and treating CVDs.

### 4.4. The Effects of Nuts on CVDs

Nuts contain several antioxidant components and possess many bioactivities [111–113]. Moderate intake of nuts also attenuated the risk factors of CVDs. An RCT found that consumption of almond which provided 15% energy significantly lowered truncal and total body fat as well as DBP in overweight or obese adults [114]. Another 6-month-long RCT demonstrated that intake of walnut remarkably reduced body weight, body mass index, waist circumference, SBP, TC, and LDL-C [115]. Moreover, supplementing type 2 diabetes participants with cashew nut considerably reduced SBP and increased HDL-C, while significant differences were not observed in body weight, body mass index, glycemic, and other lipid variables [116]. Additionally, intake of mixed nuts, including almonds, cashews, hazelnuts, pecans, Brazil nuts, macadamia nuts, pistachios, walnuts, and peanuts, could attenuate CVD risk factors by improving body weight and glucose regulation, without exerting the negative effects on lipids compared with a common carbohydrate-rich snack [117].

### 4.5. The Effects of Teas on CVDs

As the second-most consumed beverage worldwide, the nutrition value of teas is extensively investigated. Several studies found that the consumption of teas could ameliorate the risk factors of CVDs. For example, supplementation with green tea extract, which contained 1,315 mg catechins, could significantly improve lipid profile in postmenopausal women by reducing the levels of TC, LDL-C, and non-HDL-C [118]. Furthermore, a study pointed out that the consumption of phytosterol-enriched functional black tea could lower TC, LDL-C, and apolipoprotein B in mild hypercholesterolemia subjects, accompanied with the amelioration of oxidative stress [119]. Moreover, an open-label pilot study found that after daily administration of 2 g/L kosen-cha, obese subjects showed a significant reduction of body weight, BMI, waist circumferences, and serum TG levels, as well as the improvement of insulin resistance, vascular function, and cardiac hypertrophy [120].

### 4.6. The Effects of Other Plants on CVDs

Other natural products also possessed the ability to protect the health of the cardiovascular system, such as cereals, legumes, and herbs [121, 122]. For example, drinking 2 cups of *Hibiscus sabdariffa* in the morning effectively reduced blood pressure in patients with stage 1 hypertension [123]. Besides, a 3 × 3 completely randomized repeated study showed that daily consumption of 25 g or 45 g soy flour markedly lowered the levels of fasting plasma glucose, fasting insulin, insulin resistance, and DBP in postmenopausal women with prediabetes and prehypertension [124]. Additionally, using oat noodles to replace 1 or 2 meals of staple food could reduce blood pressure, and improve lipid profile by lowering TC/HDL-C and LDL-C/HDL-C ratios [125]. Hence, supplementing the consumption of *Hibiscus sabdariffa*, soy, and oat helps to reduce the risk of CVDs.

In short, clinical trials involving different conditions of subjects illustrated that some antioxidant natural products could improve cardiovascular health and reduce the risk of CVDs, which might be related to decreasing blood pressure, regulating serum lipids, lowering blood glucose, and lowering body weight.

## 5. Conclusions

As a public health problem of global concern, CVDs have attracted considerable attention. Some antioxidant natural products have been proven capable of preventing CVDs. Multiple epidemiological investigations enrolling participants from different countries, different ages, and so on, suggested that the consumption of antioxidant natural products was beneficial to reduce the risk of CVD events. Moreover, results from experimental studies showed that some natural products exerted cardiovascular-protective effects via different mechanisms of action, such as reducing blood pressure, improving the lipid profile, ameliorating oxidative stress, mitigating inflammation, and regulating gut microbiota. Furthermore, clinical trials confirmed that some antioxidant natural products could prevent and treat CVDs. Supported by current evidence, some antioxidant natural products and their active compounds could be developed into functional foods or medicine for the prevention and treatment of CVDs. In the future, the effects of more antioxidant natural products on CVDs should be evaluated to find out more cardiovascular-protective natural products, and relative bioactive components should be isolated and identified. In addition, the mechanisms of action should be elucidated further. Furthermore, special attention should be paid to the safety of relative natural products and functional foods.

## Figures and Tables

**Table 1:** The effects of antioxidant natural products on CVDs from epidemiological studies.

Plants	Components	Study type	Subjects	Results	Ref.
Fruit	NA	Cohort study	512,891 Chinese	Lowering SBP and blood glucose, reducing the risks of cardiovascular death (HR: 0.60; 95% CI, 0.54-0.67), incident major coronary event (HR: 0.66; 95% CI, 0.58-0.75), ischemic stroke (HR: 0.75; 95% CI, 0.72-0.79), and hemorrhagic stroke (HR: 0.64; 95% CI, 0.56-0.74)	[38]
Fruit	NA	Cross-sectional study	1,590 adults	Low fruit consumption was associated with increased BP in 50–59-year-old group (PR: 1.62; 95% CI, 1.09-2.41)	[43]
Fruit	Anthocyanin, flavanone	Cohort study	43.880 healthy men	Higher anthocyanin intake was inversely associated with nonfatal myocardial infarction (HR: 0.87; 95% CI, 0.75-1.00). Higher flavanone intake was inversely associated with ischemic stroke (HR: 0.78; 95% CI, 0.62-0.97).	[44]
Fruit	Flavone	Cross-sectional study	7,963 women aged ≥30 years	Inversely associated with SBP, TG, and TG/HDL-C.	[45]
Fruit	NA	Cohort study	70,047 Chinese adults with CVD or hypertension	Inversely associated with CVD mortality (HR: 0.79; 95% CI, 0.73-0.86)	[46]
Fruit	NA	Cross-sectional study	9,040 subjects aged ≥25 years	Inversely associated with CVD (OR: 0.86; 95% CI, 0.74-0.98)	[47]
Vegetable	NA	Cross-sectional study	18,757 adolescents	Consuming ≥3 servings of vegetables lowered the risk of hypertension (OR: 0.74; 95% CI, 0.58-0.94)	[35]
Vegetable	Nitrate	Cohort study	2,229 Australian aged ≥39 years	Inversely associated with CVD mortality (comparison of <69.5 mg/day intake of vegetable, 69.5-99.6 mg/day (HR: 0.53; 95% CI, 0.35-0.82), 99.7-137.8 mg/day (HR: 0.51; 95% CI, 0.32-0.80), and >137.8 mg/day (HR: 0.63; 95% CI, 0.41-0.95))	[48]
Allium vegetable	NA	Cohort study	Adult men and women	Associated with a 64% reduced risk of CVD outcomes (HR: 0.36; 95% CI, 0.18-0.71)	[39]
Nut	NA	Cohort study	16,217 participants with diabetes mellitus	Inversely associated with the total CVD incidence (HR: 0.83; 95% CI, 0.71-0.98), CHD incidence (HR: 0.80; 95% CI, 0.67-0.96), and CVD mortality (HR: 0.66; 95% CI, 0.52-0.84)	[49]
Nut	NA	3 large cohort studies	76,364 women 92,946 women 41,526 men	Inversely associated with the total CVD (HR: 0.86; 95% CI, 0.79-0.93) and CHD (HR: 0.80; 95% CI, 0.72-0.89)	[50]
Nut	NA	3 large cohort studies	34,103 men 77,815 women 80,737 women	Inversely associated with CVD (RR: 0.92; 95% CI, 0.86-0.98), CHD (RR: 0.94; 95% CI, 0.89-0.99), and stroke (RR: 0.89; 95% CI, 0.83-0.95)	[51]
Nut	NA	Cohort study	61,364 Swedish adults	Inversely associated with risk of heart failure and atrial fibrillation 1-3 times/month: heart failure (HR: 0.87; 95% CI, 0.80-0.94) and atrial fibrillation (HR: 0.97; 95% CI, 0.93-1.02) 1-2 times/week: heart failure (HR: 0.80; 95% CI, 0.67-0.97) and atrial fibrillation (HR: 0.88; 95% CI, 0.79-0.99) 3 times/week: heart failure (HR: 0.98; 95% CI, 0.76-1.27) and atrial fibrillation (HR: 0.82; 95% CI, 0.68-0.99)	[42]
Legume	NA	Cohort study	6,504 Iranian middle-aged and older people	Inversely related to the risk of CVD events in old-aged Iranians (HR: 0.66; 95% CI, 0.45-0.98) but not in middle-aged Iranians	[52]
Curry (turmeric)	Curcumin	Cross-sectional study	Individuals aged 19-64 years	Lowering blood glucose and TG levels	[37]
Fruit, vegetable	NA	Cross-sectional study	1,596 adolescents and young people in Tanzania and Uganda	Lowering the risk of hypertension (OR: 0.7; 95% CI, 0.50-0.98)	[53]
Fruit, vegetable	NA	Cross-sectional study	229 patients with primary hypertension	Lowering BP, heart rate, and BMI	[54]
Fruit, vegetable	NA	Cohort study	2,354 Ugandan newborns	Lowering BP	[21]
Fruit, vegetable	NA	Cohort study	8,997 aging subjects	Fruit intake was inversely associated with BP but vegetable intake was not	[55]
Fruit, whole grain	Fiber	Cohort study	17,007 young Mediterranean participants	Fruit (HR: 0.51; 95% CI, 0.27-0.95) or whole grain (HR: 0.43; 95% CI, 0.20-0.93) intake was inversely associated with CVD events.	[40]
Fruit, vegetable, cereal	Fiber	Cross-sectional study	18,433 American adults	Total fiber (OR: 0.62; 95% CI, 0.52-0.75), cereal fiber (OR: 0.80; 95% CI, 0.69-0.98), and vegetable fiber (OR: 0.82; 95% CI, 0.69-0.98) were inversely associated with the risk of hypertension, but fruit fiber was not	[36]
Fruit, vegetable, legume, grain, nut	Fiber	Cohort study	2,295 health professionals	Legume fiber (HR: 0.31; 95% CI, 0.15-0.65), fruit fiber (HR: 0.44; 95% CI, 0.22-0.89), and vegetable fiber (HR: 0.34; 95% CI, 0.16-0.72) were inversely associated with the CVD risks, but grain and nut fiber were not	[41]

*Note*. NA: not available; CVD: cardiovascular disease; CHD: coronary heart disease; HR: hazard ratio; PR: prevalence ratio; OR: odds ratio; RR: relative ratio; CI: confidence interval; BW: body weight; BP: blood pressure; SBP: systolic blood pressure; TG: triglyceride; BMI: body mass index; HDL-C: high-density lipid protein cholesterol.

**Table 2:** The effects of antioxidant natural products on CVDs from experimental studies.

Plants	Components	Study type	Subjects	Dose & Time	Effects and mechanisms	Ref.
Winged bean seed	Peptide	*In vitro* *In vivo*	ACE and SD rats	1 mM peptides, 3 h; 150 and 300 mg/kg BW, 24 h	Inhibiting ACE activity Lowering BP	[61]
*Solanum macrocarpon*	Polyphenols	*In vitro* *In vivo*	SHRs	100 and 500 mg/kg BW	Inhibiting ACE/renin activities Lowering DBP and heart rate	[62]
*Citrus paradisi* and *Ocimum sanctum*	Epigallocatechin gallate and quercetin	*In vivo*	SD rats	2 g dried ground material in 200 mL water, 4 months	Reducing BP (renin and angiotensinogen↓) and reducing renal TG accumulation and lipid/protein oxidation (*Citrus paradisi*) Reducing BP via other mechanisms (*Ocimum sanctum*)	[63]
Pigeon pea	Protein	*In vitro* *In vivo*	ACE and SHRs	100 mg/kg BW, 24 h	Inhibiting ACE/renin activities and scavenging free radicals Lowering BP	[24]
*Ficus deltoidea* var. Kunstleri	NA	*In vivo*	SHRs	500, 800, 1000, and 1300 mg/kg BW, 4 weeks	Lowering BP (ACE, angiotensin, aldosterone↓, and eNOS↑) and improving antioxidant capacity	[97]
*Pueraria lobata*	Puerarin	*In vivo*	SHRs	40 and 80 mg/kg, 9 weeks	Lowering BP (eNOS, NO, and cGMP↑)	[98]
White mulberry fruit	Polysaccharides	*In vitro* *In vivo*	Mesenteric artery and endothelial cells; SD rats and SHRs	0.5 mg/mL; 5 mg/kg, 5 min	Inducing endothelium-dependent relaxation in rat mesenteric arteries and NO production in endothelial cells Lowering mean arterial BP	[67]
Grape seed	Polyphenols	*In vivo*	Hypertensive rats	375 mg/kg	Lowering BP (eNOS and Sirtuin-1↑)	[68]
*Morus alba*	Rutin	*In vitro* *In vivo*	Mesenteric arteries; wild-type and eNOS-deficient mice	8 mg/mL; 100, 200, and 400 mg/kg	Inducing endothelial vasorelaxation via a NO-dependent pathway Decreasing BP in wild-type mice, not in eNOS-deficient mice	[66]
*Phyllanthus niruri*	NA	*In vitro* *In vivo*	Endothelium-intact/denuded aorta rings; SHRs	0.125-4 mg/mL; 1000 mg/kg BW, 2 weeks	Inducing vasorelaxation on endothelium-intact aorta rings Decreasing BP	[99]
*Scutellaria baicalensis* Georgi	Baicalin	*In vitro* *In vivo*	Thoracic aortas; SHRs	0.1 mg/mL; 10, 50, 100, and 200 mg/kg BW, 0, 30, 60, 90, and 120 min	Relaxing SHR aortas in an endothelium-independent manner Reducing BP	[100]
*Heliotropium strigosum*	Polyphenols	*In vivo*	Diabetic rabbits	21 days	Improving lipid profile (TC, TG, and LDL-C↓) and lowering blood glucose	[25]
Mung bean sprouts	NA	*In vivo*	SD rats	1 mL/200 g BW, 8 weeks	Lowering BP and improving lipid profile (LDL-C↓)	[72]
Red dragon fruit	NA	*In vivo*	SD rats	4 weeks	Improving lipid profile (TC, TG, and LDL-C↓) and lowering blood glucose	[73]
Red dragon fruit peel	NA	*In vivo*	Hyperlipidemia male mice	50, 100, 150, and 200 mg/kg BW, 30 days	Improving lipid profile (TC, TG↓, and HDL-C↑)	[74]
*Citrus maxima*	NA	*In vivo*	Wistar rats	300 and 600 mg/kg BW, 14 days	Improving lipid profile (TC, TG↓, and HDL-C↑), lowering blood glucose, and increasing BW	[75]
Bitter melon	*β*-Sitosterol	*In vivo*	Hyperglycemia rats	71.1 mg, 4 weeks	Improving lipid profile (TC, TG, LDL-C, fecal cholesterol secretion, cholesterol absorption↓, and HDL-C↑) and lowering blood glucose	[76]
Dried chokeberry	Anthocyanins	*In vivo*	SHRs	50 mg/kg and 4 weeks	Ameliorating oxidative stress (TBARS↓ and FRAP↑) and lowering SBP and pulse pressure	[26]
Sweet cherry	Polyphenols	*In vivo*	Wistar rats	5% and 10% (*w*/*w*) in food (fruits); 1% and 3% (*w*/*w*) in food (leaves), 12 weeks	Decreasing BW gain, ameliorating oxidative stress (SOD, GPx, CAT↑, and TBARS↓), and improving lipid profile (LDL-C+VLDL-C↓)	[82]
Wild rice	NA	*In vivo*	Hyperlipidemic rats	NA and 8 weeks	Ameliorating oxidative stress (TAC, SOD↑, and MAD↓), improving lipid profile (TG and TC↓), and mitigating inflammation (CRP and TNF-*α*↓)	[80]
*Sambucus nigra* L.	Polyphenols	*In vivo*	Wistar rats	0.046 g/kg BW, 8 weeks	Ameliorating oxidative stress (TAC↑), lowering BP, and improving lipid profile (HDL-C↑)	[81]
*Nepeta deflersiana*	NA	*In vivo*	Wistar rats	50 and 100 mg/kg BW, 25 days	Attenuating myocardial injuries, mitigating inflammation (TNF-*α*, IL-6, and IL-10↓), and improving oxidative stress (CAT, SOD, NO↑, and MDA↓)	[29]
Spinach	Nitrate	*In vivo*	Swiss-Kunming mice	15, 30, and 60 mg/kg of nitrate, 28 days	Mitigating inflammation (CRP, TNF-*α*, and IL-6↓) and improving vascular endothelial function (NO↑ and endothelin-1↓), lipid profile (TC, TG, LDL-C↓, and HDL-C↑), and insulin resistance	[88]
*Zygophyllum album* roots	NA	*In vivo*	Wistar rats	400 mg/kg BW, 60 days	Attenuating myocardial injuries, improving oxidative stress (MDA, PC↓, CAT, SOD, and GPx↑), and mitigating inflammation (TNF-*α*, IL-1*β*, IL-6, and nuclear factor-kappa B↓)	[84]
*Spinacia oleracea*	Lutein	*In vivo*	Wistar rats	100, 200, and 300 mg/kg BW	Ameliorating myocardial necrosis via mitigating inflammation (TNF-*α*, IL-1*β* and IL-6↓)	[89]
*Antidesma bunius*	NA	*In vivo*	SD rats	0.38, 0.76, and 1.52 g/kg, 12 weeks	Improving oxidative stress (MDA↓) and mitigating inflammation (TNF-*α*, IL-6, VCAM-1, and MCP-1↓)	[90]
Rice bran	Protein	*In vivo*	SD rats	250 and 500 mg/kg, 6 weeks	Lowering BP (ACE↓, NO, and eNOS↑) and reducing arterial stiffening, vascular remodeling, and oxidative stress (SOD and MDA↓)	[83]
*Polygoni multiflori* Radix	2,3,5,4′-Tetrahydroxy-stilbene-2-O-beta-D-glucoside	*In vivo*	ApoE(-/-) mice	1.125 mg/g, 8 weeks	Inhibiting atherosclerotic plaque formation, improving lipid profile (TG and ox-LDL↓), mitigating inflammation (TNF-*α*, IL-6, VCAM-1, and ICAM-1↓), and regulating gut microbiota composition (*Firmicutes*/*Bacteroidetes*, *Akkermansia*↑, *Proteobacteria*, *Tenericutes*, and *Helicobacter pylori*↓)	[28]
Wasabi	Allyl isothiocyanate	*In vivo*	Wistar rats	5% (*w*/*w*) in food, 8 weeks	Regulating gut microbiota composition to prevent the development of hypertension (*Allobaculum*, *Sutterella*, *Uncl. S247*, *Uncl. Coriobacteriaceae*, and *Bifidobacterium*↑)	[93]
*Lycium ruthenicum* Murray	Anthocyanins	*In vivo*	C57BL/6 mice	200 mg/kg, 12 weeks	Improving oxidative stress (TAC, SOD, GPx↑, and MDA↓) and inflammation (TNF-*α*, IL-6, and IL-1*β*↓), regulating gut microbiota (*Barnesiella*, *Alistipes*, *Eisenbergiella*, *Coprobacter*, and *Odoribacter*↑), and increasing SCFA in cecal and feces	[94]
Tea	Polyphenols	*In vivo*	ApoE(-/-) mice	1.6, 0.8, and 0.4 g/L tea polyphenols in drinking water	Lowering TC and LDL-C, decreasing the plaque area/lumen area, and promoting the proliferation of the intestinal *Bifidobacteria*	[95]
Berry mixture	Polyphenols	*In vivo*	Dahl salt-sensitive rats	2 g, 9 weeks	Mitigating changes in the microbiota composition caused by the high-salt diet (phylum *Bacteroidetes*↑, *Firmicutes*, and *Proteobacteria*↓)	[96]

*Note*. NA: not available; SHRs: spontaneously hypertensive rats; SD rats: Sprague-Dawley rats; BW: body weight; *w*/*w*: weight in weight; BP: blood pressure; SBP: systolic blood pressure; DBP: diastolic blood pressure; ACE: angiotensin-1 converting enzyme; NO: nitric oxide; eNOS: endothelial nitric oxide synthase; cGMP: cyclic guanosine monophosphate; TC: total cholesterol; TG: triglyceride; LDL-C: low-density lipoprotein cholesterol; HDL-C: high-density lipoprotein cholesterol; VLDL-C: very-low-density lipoprotein cholesterol; ox-LDL: oxidized low-density lipoprotein; TAC: total antioxidant capacity; FRAP: ferric ion-reducing antioxidant power; MDA: malondialdehyde; PC: protein carbonyls; TBARS: thiobarbituric acid reactive substances; SOD: superoxide dismutase; GPx: glutathione peroxidase; GR: glutathione reductase; CAT: catalase; TNF-*α*: tumor necrosis factor *α*; CRP: C reactive protein; IL-1*β*: interleukin-1*β*; IL-6: interleukin-6; IL-10: interleukin-10: VCAM-1: vascular cell adhesion molecule 1; ICAM-1: intercellular adhesion molecule 1; MCP-1: monocyte chemotactic protein 1; SCFA: short-chain fatty acids.

**Table 3:** The effects of antioxidant natural products on CVDs from clinical studies.

Plant types	Components	Study type	Subjects	Dose and time	Outcomes	Ref.
*Fruits*						
Guava	NA	RCT	45 healthy students	400 g/day, 6 weeks	Lowering BP, TC, TG, and LDL-C	[101]
Soursop	NA	RCT	143 hypertensive subjects	2 × 100 g/day, 3 months	Lowering BP	[102]
Orange juice	Hesperidin and naringin	Controlled nonrandomized clinical study	10 healthy women	300 mL/day, 2 months	Improving LDL-C, blood glucose, insulin sensitivity, and gut microbiota metabolism	[103]
Haskap berry	Anthocyanin	Cross-over study	20 adults aged 62-81 years	400 mg anthocyanins	Lowering BP and improving episodic memory	[104]
Cherry juice	Anthocyanin	Pilot cross-over study	6 young and 7 old adults	300 mL or 100 mL, 3 times	Lowering BP and heart rate	[126]
Pomegranate extract	Polyphenols	RCT	55 subjects without any symptomatic disease	Containing 210 mg punicalagins, 328 mg other pomegranate polyphenols, and 0-37 mg anthocyanins, 8 weeks	Lowering SBP	[127]
Plum juice	Anthocyanins	Pilot cross-over dose-timing study	12 older (65+ years) and 12 younger (18-45 years) adults	300 mL or 100 mL, 3 times	Reducing BP and cardiovascular responses	[128]
Noni and chokeberry juices	NA	RCT	88 young adults	Noni juice 30 mL; chokeberry juice 200 mL	Lowering SBP, DBP, heart rate, and blood glucose (noni juice) Slightly lowering DBP (chokeberry juice)	[129]
*Vegetables*						
Tomato extract	NA	RCT	65 patients with hypertension and a high risk of CVD	213 mg/day, 4 weeks	Lowering DBP and mean arterial pressure	[105]
Beetroot juice	Nitrate	Open-label cross-over study	17 patients with chronic kidney disease	Containing 300 mg nitrate, 4 hours	Lowering peripheral BP and mean arterial pressure	[130]
Beetroot juice	Nitrate	Double-blind cross-over study	20 subjects with treated yet uncontrolled hypertension	Containing 12.9 mmol nitrate, 7 days	Increasing plasma nitrite and reducing BP	[131]
Beetroot juice	Nitrate	Feasibility trial	40 hypertensive pregnant women	70 mL/day, 8 days	Lowering DBP	[106]
*Sateria palmifolia*	NA	Quasiexperiment	10 pregnant women	NA	Lowering BP	[132]
Eggplant powder	NA	RCT	100 stressed participants with normal-high BP or stage 1 hypertension	1.2 g/day, 12 weeks	Improving BP and psychological state	[107]
Beetroot, rocket salad and spinach	Nitrate	Semirandomized cross-over study	11 men and 7 women	Each group containing 800 mg nitrate	Lowering BP	[133]
*Spices*						
*Satureja hortensis* L.	NA	RCT	47 patients with metabolic syndrome	450 mg/day, 10 weeks	Lowering TC, TG, and LDL-C and increasing HDL-C	[109]
Cardamom	NA	RCT	80 overweight and obese prediabetic women	3 g, 2 months	Lowering TC and LDL-C	[108]
Garlic and cumin	NA	RCT	75 patients with T2DM	Garlic powder: 300 mg/three times a day; cumin extract: 100 mg/twice a day, 2 months	Lowering BP	[110]
Cinnamon, cardamom, saffron, and ginger	NA	RCT	204 patients with T2DM	3 g cinnamon, 3 g cardamom, 1 g saffron, and 3 g ginger in a glass of black tea, respectively, 8 weeks	All showed potent effects on controlling BP and improving endothelial function	[33]
*Nuts*						
Almond	NA	RCT	86 overweight or obese adults	15% energy from almond, 12 weeks	Lowering truncal and total body fat as well as DBP	[114]
Walnuts	NA	RCT	211 participants	30 g/day, 3 months	Lowering BP	[134]
Walnuts	NA	RCT	100 overweight and obese participants	15% energy from walnut, 6 months	Reducing BW, BMI, waist circumference, SBP, TC, and LDL-C	[115]
Walnuts	*α*-Linolenic acid	RCT	42 adults at cardiovascular risk	57–99 g/day, 6 weeks	Regulating gut microbiota	[135]
Cashew nut	NA	RCT	300 Asian Indians with T2DM	30 g/day, 12 weeks	Decreasing SBP and increasing HDL-C	[116]
Cashew nut	NA	RCT	42 adults	42 g/day, 4 weeks	No effect on risk factors of CVD	[136]
Mixed nuts	NA	RCT	48 overweight and obese adults	250 kcal, 4 and 8 weeks	Improving BW and glucose regulation	[117]
*Teas*						
Green tea	Catechin	RCT	1,075 postmenopausal women	Containing 1,315 mg catechins, 6 and 12 months	Lowering TC, LDL-C, and non-HDL-C	[118]
Kosen-cha	Catechin	Open-label pilot study	6 obese subjects	5 g/L, 12 weeks	Lowering BW, BMI, waist circumferences, and serum TG levels and improving insulin resistance, vascular function, and cardiac hypertrophy	[120]
Goishi tea	Polyphenols	RCT	77 subjects	Containing 122 mg of polyphenols, 12 weeks	Increasing HDL-C and lowering TG	[137]
Black tea	Phytosterol	RCT	Subjects with mild hypercholesterolemia	Phytosterol-enriched functional black tea, 4 weeks	Lowering TC, LDL-C, and apolipoprotein B and improving oxidative stress	[119]
*Others*						
*Hibiscus sabdariffa*	NA	Cross-over study	25 men with 1% to 10% CVD risk	250 mL, 0, 2, and 4 h	Reducing endothelial dysfunction and CVD risk	[138]
*Hibiscus sabdariffa*	NA	RCT	46 patients with stage 1 hypertension	2 cup/morning, 1 month	Reducing BP	[123]
Chamomile	NA	RCT	50 diabetic patients	200 mL/day, 4 weeks	Lowering TC, LDL-C, and creatinine	[139]
Soy flour	NA	3 × 3 completely randomized repeated study	75 postmenopausal women with prediabetes and prehypertension	25 and 45 g/day, 12 weeks	Lowering fasting plasma glucose, fasting insulin, insulin resistance, and DBP	[124]
Navy beans and rice bran	NA	Pilot RCT	38 children with abnormal cholesterol	17.5 g/day cooked navy bean powder; 15 g/day heat-stabilized rice bran; 9 g/day navy beans and 8 g/day rice bran, 4 weeks	Modulating the plasma metabolome and reducing CVD risk	[140]
Oat noodles	NA	RCT	84 healthy and mild hypercholesterolemic subjects	100 g/day (replacing 1 or 2 meals of staple food), 10 weeks	Reducing TC/HDL-C and LDL-C/HDL-C ratios and blood pressure	[125]
Green coffee bean	NA	RCT	Patients with the metabolic syndrome	400 mg twice/day, 8 weeks	Reducing SBP, insulin resistance, and abdominal obesity and inhibiting appetite	[141]

*Note*. NA: not available; RCT: randomized controlled trial; CVD: cardiovascular disease; T2DM: type 2 diabetes mellitus; BW: body weight; BMI: body mass index; BP: blood pressure; SBP: systolic blood pressure; DBP: diastolic blood pressure; TC: total cholesterol; TG: triglyceride; LDL-C: low-density lipoprotein cholesterol; HDL-C: high-density lipoprotein cholesterol.
